# Qualitative and Quantitative Analysis of *Rhizoma Smilacis glabrae* by Ultra High Performance Liquid Chromatography Coupled with LTQ Orbitrap^XL^ Hybrid Mass Spectrometry

**DOI:** 10.3390/molecules190710427

**Published:** 2014-07-17

**Authors:** Shao-Dan Chen, Chuan-Jian Lu, Rui-Zhi Zhao

**Affiliations:** 1 The Second College of Clinic Medicine, Guangzhou University of Chinese Medicine, Guangzhou 510000, China; 2 Guangdong Provincial Hospital of Chinese Medicine, Guangzhou 510000, China

**Keywords:** *Rhizoma Smilacis glabrae*, UHPLC-ESI/LTQ-Orbitrap-MS, qualitative analysis, quantitative analysis

## Abstract

*Rhizoma Smilacis glabrae*, a traditional Chinese medicine (TCM) as well as a functional food, has been commonly used for detoxification treatments, relieving dampness and as a diuretic. In order to quickly define the chemical profiles and control the quality of *Smilacis glabrae*, ultra high performance liquid chromatography coupled with electrospray ionization hybrid linear trap quadrupole orbitrap mass spectrometry (UHPLC-ESI/LTQ-Orbitrap-MS) was applied for simultaneous identification and quantification of its bioactive constituents. A total of 56 compounds, including six new compounds, were identified or tentatively deduced on the basis of their retention behaviors, mass spectra, or by comparison with reference substances and literature data. The identified compounds belonged to flavonoids, phenolic acids and phenylpropanoid glycosides. In addition, an optimized UHPLC-ESI/LTQ-Orbitrap-MS method was established for quantitative determination of six marker compounds from five batches. The validation of the method, including linearity, sensitivity (LOQ), precision, repeatability and spike recoveries, was carried out and demonstrated to be satisfied the requirements of quantitative analysis. The results suggested that the established method would be a powerful and reliable analytical tool for the characterization of multi-constituent in complex chemical system and quality control of TCM.

## 1. Introduction

The rhizome of *Smilacis glabrae* Roxb (family Smilacaceae) is a well-known traditional Chinese medicine (TCM) with great medicinal values. It is officially listed in the Chinese Pharmacopoeia and has been widely used for detoxification treatments, relieving dampness and as a diuretic [1]. It was also consumed as a functional food. People in China like to use it to boil soup or tea for clearing damp. Besides, it is one of the main ingredients of turtle jelly (Gui-ling-gao), a traditional functional food popular in Southern China and Hong Kong. Phytochemical studies have shown the presence of abundant compounds in *S. glabrae*, such as flavonoids, phenolic acids and phenylpropanoid glycosides [2,3], among which flavonoids were considered to be the primary bioactive constituents of the herbal medicine. Astilbin, neoastilbin, isoastilbin, neoisoastilbin, engeletin and isoengeletin were considered as marker constituents included in *S. glabrae*. These six flavonoids were reported to possess various biological activities, involving anti-inflammatory, antioxidative, antibacterial and antitumor properties [4,5,6,7,8,9,10]. Some analytical methods have been used for qualitative or quantitative analysis of some of these bioactive constituents in *S. glabrae*. Li *et al.* identified the main constituents in *Rhizoma Smilacis glabrae* by means of UHPLC-DAD-MS [3]. Chen *et al.* established an HPLC method for determination of five compounds in *Rhizoma Smilacis glabrae* [11]. Although these methods have made significant contributions to the studies of the quality control of *Smilacis glabrae*, they have limitations, such as taking a long time to perform or being either qualitative or quantitative. Less effort has been dedicated to further characterize minor new components or the rapid determination of active components, so a new method is required to address the limitations of the previous techniques.

The present work aimed at developing a rapid and simple UHPLC-ESI-MS method for analyzing and discovering minor new constituents, and quantifying the active components in *Smilacis glabrae*. The advantages of this method comprised high-speed detection, excellent peak shapes, and less solvent usage. With the new method it took less than 10 min to detect 56 compounds of *Smilacis glabrae*, including six new compounds. Further, six marker flavonoids were quantitatively determined in negative ionization mode and five batches of *Smilacis glabrae* were analyzed for assessment of quality consistence. This is the first time for determination of multiple components in *Smilacis glabrae* using UHPLC-ESI/LTQ-Orbitrap-MS.

## 2. Results and Discussion

### 2.1. Optimization of Chromatographic Conditions

To improve the resolution and sensitivity of the analysis but reduce the analytical time, the mobile phase system was optimized. To inhibit ionization of the acidic ingredients in *Smilacis glabrae* extract, formic acid was added to the mobile phase. Two mobile phase systems, methanol-aqueous solution and acetonitrile-aqueous solution were compared. Both negative and positive modes were examined. Generally, in positive mode, low abundance of [M+H]^+^, [M+NH_4_]^+^ ions and few product ions were observed, while, in negative ion mode, a series of [M-H]^−^ ions and/or adduct ions ([M+HCOOH-H]^−^) appeared with sufficient abundance. Thus the negative ion mode was chosen and the [M-H]^−^/([M+HCOOH-H]^−^) ions were further subjected to LC-MS^n^ analysis.

### 2.2. Identification of Chemical Constituents in Smilacis glabrae Extract

**Figure 1:**
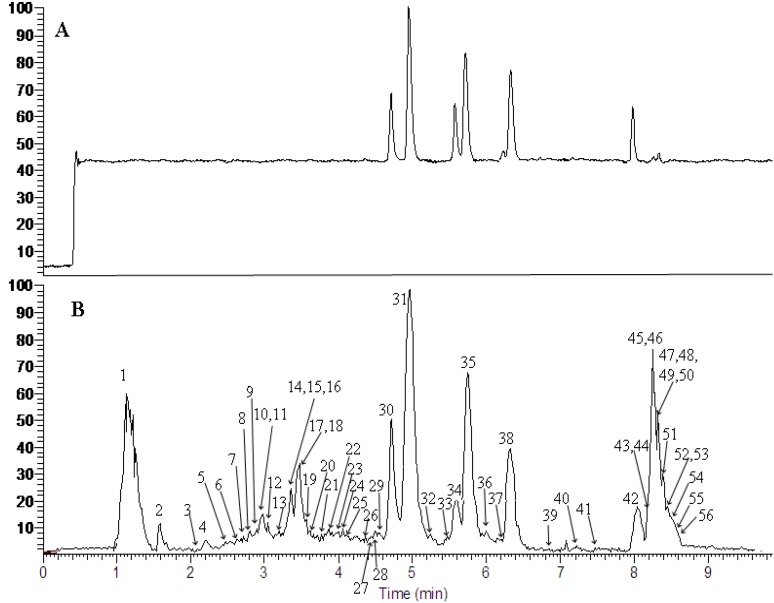
UHPLC-(-) ESI-MS total ion chromatograms of a mixture of six standards (**A**) and the extract of *Smilacis glabrae* (**B**).

The reference standards and *Smilacis glabrae* sample were analyzed by using the optimized UHPLC-ESI-MS^n^ method. The TIC chromatograms of the six reference standards and the extract of *Smilacis glabrae* in negative ESI mode were shown in Figure 1. Fifty six peaks were observed. The MS data showed high precision with all the mass accuracies within 5 ppm. For most of the constituents, a [M-H]^−^ peak was observed. Due to the use of formic acid in mobile phase, there were additional ions of [M+46-H]^−^ corresponding to [M+HCOOH-H]^−^ in negative ion mode. These results provided valuable information for confirming accurate molecular weights and composition of the constituents. The 56 compounds including six new ones were tentatively identified on the basis of their retention behaviors, accurate molecular weight and MS^n^ fragment data, or by comparison with reference standards or literature data (chemical structures of the compounds corresponding to the peaks shown in Figure 1 below can be found in Figure 1 in the Supplementary). The corresponding quasimolecular ions and their fragment ions in the MS^n^ spectra are listed in Table 1.

**Figure 2:**
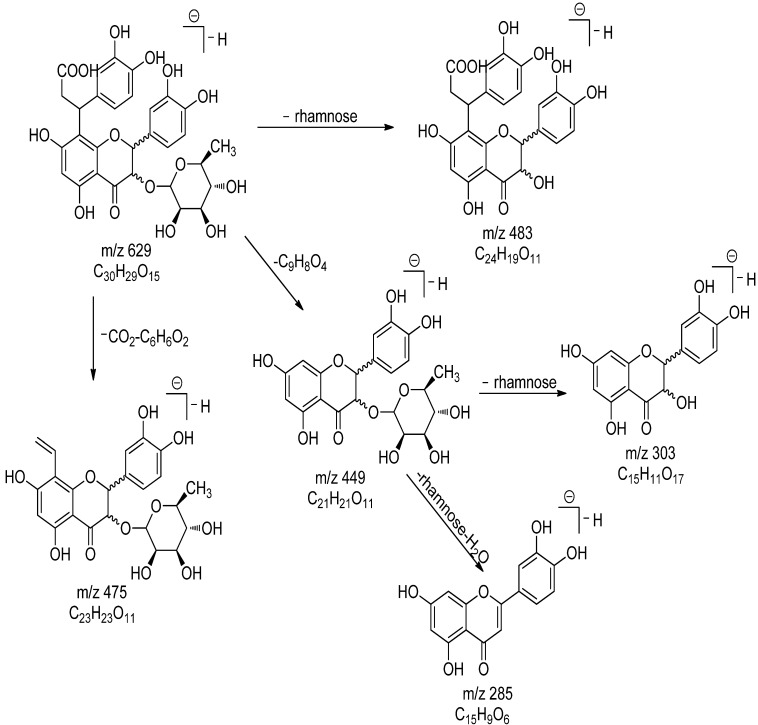
Proposed fragmentation pathways for compounds **19**, **21**, **25** and **29**.

The identified compounds can be classified into three classes, namely flavonoids, phenolic acids and phenylpropanoid glycosides. Four flavanonol isomers (compounds **30**, **31**, **34** and **35**) were unambiguously identified by the same deprotonated ions at *m/z* 449 (C_21_H_21_O_11_) and the same product ions at *m/z* 303 and *m/z* 285, and they could be distinguished through their UV absorption and elution order when compared to reference standards. Neoastilbin (**30**) with 2*S*,3*S* configuration and astilbin (**31**) with 2*R*,3*R* configuration had the same UV_max_ absorption at 290 nm, while neoisoastilbin (**34**) with 2*S*,3*R* configuration and isoastilbin (**35**) with 2*R*,3*S* configuration had the same UV absorption at 295–296 nm (see Figure 2 in the Supplementary), the latter caused a red shift of 5–6 nm, and the elution order of the four flavanonol isomers were 2*S*,3*S* > 2*R*,3*R* > 2*S*,3*R* > 2*R*,3*S*. The four flavanonols were the main constituents of *S. glabrae*. To our surprise, compounds **19**, **21**, **25** and **29** had the same deprotonated ions at *m/z* 629 (C_30_H_29_O_15_) and the same fragment ions (Figure 2), which demonstrated they were also diastereomers. In the MS^2^ spectra, the product ions at *m/z* 449 [M-H-C_9_H_8_O_4_] and *m/z* 303 [M-H-C_9_H_8_O_4_-rhamnose] suggested the four diastereomers were the derivatives of the four configurationally different astilbins. In addition, two prominent MS^2^ product ions were observed at *m/z* 475 and *m/z* 483, respectively, for the neutral loss of CO_2_ + C_6_H_6_O_2_ and for the loss of a rhamnose, which indicated they had the same substituent group and substituent site. The four isomers could also be distinguished through their UV absorption. Compounds **19** and **21** had the same UV absorption at 289 nm, while compounds **25** and **29** had the same UV absorption at 295 nm (see Figure 2 in the Supplementary), which indicated that compounds **19** and **21** had the 2*S*,3*S* or 2*R*,3*R* configuration, while compounds **25** and **29** had the 2*S*,3*R* or 2*R*,3*S* configuration. As the elution order was 2*S*,3*S* > 2*R*,3*R* > 2*S*,3*R* > 2*R*,3*S*, thus compounds **19**, **21**, **25** and **29** were tentatively identified as 8-[β-(3,4-dihydroxyphenyl)-α-carboxyl-3-oxopropyl]-substituted neoastilbin, 8-[β-(3,4-dihydroxyphenyl)-α-carboxyl-3-oxopropyl]-substituted astilbin, 8-[β-(3,4-dihydroxyphenyl)-α-carboxyl-3-oxopropyl]-substituted neoisoastilbin and 8-[β-(3,4-dihydroxyphenyl)-α-carboxyl-3-oxopropyl]-substituted isoastilbin, respectively. Similarly, compounds **38** and **42** were unambiguously identified as engeletin (**38**) and isoengeletin (**42**) based on reference standards, and compounds **24** and **28** were tentatively identified as 8-[β-(3,4-dihydroxyphenyl)-α-carboxyl-3-oxopropyl]-substituted engeletin and 8-[β-(3,4-dihydroxyphenyl)-α-carboxyl-3-oxopropyl]-substituted isoengeletin, respectively (Figure 3 in the Supplementary). Compounds **19**, **21**, **24**, **25**, **28** and **29** were identified as new compounds, but their absolute configurations could not be determined.

### 2.3. Method Validation of the Quantitative Analysis

The calibration curves, linear ranges, limit of quantification (LOQ) and repeatability of six analytes were performed using the above-developed UHPLC-ESI-MS method (Table 2). Reasonable correlation coefficient values (*r*^2^ ≥ 0.9981) indicated good correlations between investigated standards concentrations and their peak areas within the ranges tested. The ranges of LOQ for all the analytes were from 0.011 to 0.067 μg/mL, respectively. The repeatability present as RSD (*n* = 6) was between 1.77% and 2.37% of the 6 analytes. The overall intra- and inter-day precisions (RSD) of the six analytes were in the range from 1.03% to 3.19%, and 0.76% to 3.91% (Table 2), respectively. The developed method had good accuracy with the RSD of the recoveries were between 1.49% and 4.73% (Table 2). Therefore, the results demonstrated that the UHPLC-ESI-MS method was sensitive, precise, and accurate enough for quantitative evaluation of *Smilacis glabrae*.

**Table 1:** Identification of the chemical constituents of *Smilacis glabrae* by UHPLC-ESI-MS^n^ analysis.

Peak No.	*t*_R_ (min)	Selected Ion		Observed Mass (*m/z*)		Calculated Mass (*m/z*)			Formula			MS/MS Fragmentation Patterns			Identifieation		
1 ^a^	1.15	[M-H]^−^		173.0457		173.0450			C_7_H_9_O_5_			173→155, 129, 111			shikimic acid		
2	1.62	[M-H]^−^		117.0195		117.0188			C_4_H_5_O_4_			117→99, 73			succinic acid		
3	2.32	[M-H]^−^		359.0984		359.0978			C_15_H_19_O_10_			359→197, 182			syringic acid-4-*O*-β-d-glucopyranoside		
4	2.34	[M+COOH]^−^		255.0512		255.0505			C_11_H_11_O_7_			255→209, 193, 179, 165			3,4-dihydroxy-5-methoxycinnamic acid		
5	2.47	[M+COOH]^−^		345.1191		345.1186			C_15_H_21_O_9_			345→299			rhodioloside		
6 ^a^	2.70	[M-H]^−^		153.0194		153.0188			C_7_H_5_O_4_			153→109			protocatechuic acid		
7	2.91	[M+COOH]^−^		197.0458		197.0450			C_9_H_9_O_5_			197→153			syringic acid		
8	2.97	[M-H]^−^		387.1296		387.1291			C_17_H_23_O_10_			387→207, 177			3-(β-d-glucopyranosyloxy)-1-(4-hydroxy-3,5-dimethoxyphenyl)-1-propanone		
9	3.08	[M-H]^−^		577.1346		577.1346			C_30_H_25_O_12_			577→559, 451, 425, 407, 289			procyanidin B		
10 ^a^	3.13	[M-H]^−^		289.0720		289.0712			C_15_H_13_O_6_			289→271, 245, 205,179,151			catechin		
11	3.19	[M-H]^−^		239.0564		239.0556			C_11_H_11_O_6_			239→221, 195, 179, 177, 149			syringic acid acetate		
12	3.36	[M-H]^−^		315.1074		315.1080			C_14_H_19_O_8_			315→153			3,4-dihydroxyphenethyl glucoside		
13 ^a^	3.45	[M-H]^−^		469.1141		469.1135			C_24_H_21_O_10_			469→315, 289			(2 *R*,3*S*)-8-[β-(3,4-dihydroxyphenyl)-α-carboxyl-3-oxopropyl]-substituted catechin		
14 ^a^	3.55	[M-H]^−^		335.0777		335.0767			C_16_H_15_O_8_			335→291, 179, 135			3-*O*-caffeoylshikimic acid		
15 ^a^	3.58	[M-H]^−^		561.1397		561.1397			C_30_H_25_O_11_			561→543, 435, 289			3',4',5,7-tetrahydroxyflavan(4°8)-3,3',4',5,7-pentahydroxyflavan		
16 ^a^	3.61	[M-H]^−^		289.0722		289.0712			C_15_H_13_O_6_			289→271, 245, 205, 179, 151			epicatechin		
17 ^a^	3.74	[M-H]^−^		335.0777		335.0767			C_16_H_15_O_8_			335→291, 179, 135			4-*O*-caffeoylshikimic acid		
18 ^a^	3.76	[M-H]^−^		179.0350		179.0344			C_9_H_7_O_4_			179→161, 135			caffeic acid		
19 ^b^	3.93	[M-H]^−^		629.1514		629.1506			C_30_H_29_O_15_			629→483, 475, 449, 303, 285			8-[β-(3,4-dihydroxyphenyl)-α-carboxyl-3-oxopropyl]-substituted neoastilbin		
20	4.04	[M-H]^−^		465.1041		465.1033			C_21_H_21_O_12_			465→421, 297			4-*O*-β-d-(6-*O*-gentisoylglucopyranosyl)-vanillic acid		
21 ^b^	4.20	[M-H]^−^		629.1514		629.1506			C_30_H_29_O_15_			629→483, 475, 449, 303, 285			8-[β-(3,4-dihydroxyphenyl)-α-carboxyl-3-oxopropyl]-substituted astilbin		
22	4.23	[M-H]^−^		339.0721		339.0716			C_15_H_15_O_9_			339→193			smiglanin		
23 ^a^	4.39	[M-H]^−^		335.0777		335.0767			C_16_H_15_O_8_			335→291, 179, 135			5-*O*-caffeoylshikimic acid		
24 ^b^	4.44	[M-H]^−^		613.1565		613.1557			C_30_H_29_O_14_			613→467, 459, 433, 287			8-[β-(3,4-dihydroxyphenyl)-α-carboxyl-3-oxopropyl]-substituted engeletin		
25 ^b^	4.56	[M-H]^−^		629.1514		629.1506			C_30_H_29_O_15_			629→483, 475, 449, 303, 285			8-[β-(3,4-dihydroxyphenyl)-α-carboxyl-3-oxopropyl]-substituted neoisoastilbin		
26	4.84	[M-H]^−^		301.0354		301.0348			C_15_H_9_O_7_			301→283, 255, 215, 175, 151			quercetin		
27	4.97	[M+COOH]^−^		435.1297		435.1291			C_21_H_23_O_10_			435→389, 227,195			polydatin		
28 ^b^	5.06	[M-H]^−^		613.1565		613.1557			C_30_H_29_O_14_			613→467, 459, 433, 287			8-[β-(3,4-dihydroxyphenyl)-α-carboxyl-3-oxopropyl]-substituted isoengeletin		
29 ^b^	5.12	[M-H]^−^		629.1514		629.1506			C_30_H_29_O_15_			629→483, 475, 449, 303			8-[β-(3,4-dihydroxyphenyl)-α-carboxyl-3-oxopropyl]-substituted isoastilbin		
30 ^c^	5.29	[M-H]^−^		449.1099		449.1084			C_21_H_21_O_11_			449→303, 285			neoastilbin		
31 ^c^	5.63	[M-H]^−^		449.1099		449.1084			C_21_H_21_O_11_			449→303, 285			astilbin		
32 ^a^	5.72	[M-H]^−^		193.0511		193.0501			C_10_H_9_O_4_			193→178, 161, 134			ferulic acid		
33 ^a^	6.10	[M-H]^−^		303.0513		303.0505			C_15_H_11_O_7_			303→285, 177, 125			taxifolin		
34 ^c^	6.55	[M-H]^−^		449.1099		449.1084			C_21_H_21_O_11_			449→303, 285			neoisoastilbin		
35 ^c^	6.81	[M-H]^−^		449.1099		449.1084			C_21_H_21_O_11_			449→303, 285			isoastilbin		
36	6.86	[M-H]^−^		243.0665		243.0657			C_14_H_11_O_4_			243→225, 201, 199, 175			piceatannol		
37	7.27	[M-H]^−^		433.1149		433.1135			C_21_H_21_O_10_			433→287, 269			neoengeletin		
38 ^c^	7.43	[M-H]^−^		433.1149		433.1135			C_21_H_21_O_10_			433→287, 269			engeletin		
39 ^a^	7.49	[M-H]^−^		359.0771		359.0767			C_18_H_15_O_8_			359→341, 291, 239, 197			rosmarinic acid		
40	7.53	[M-H]^−^		433.1149		433.1135			C_21_H_21_O_10_			433→287,269			neoisoengeletin		
41	8.16	[M-H]^−^		693.2029		693.2031			C_32_H_37_O_17_			693→517, 337			helonioside A		
42 ^c^	8.20	[M-H]^−^		433.1149		433.1135			C_21_H_21_O_10_			433→287,269			isoengeletin		
43 ^a^	8.23	[M-H]^−^		451.1038		451.1029			C_24_H_19_O_9_			451→341			cinchonain Ia		
44	8.25	[M-H]^−^		693.2029		693.2031			C_32_H_37_O_17_			693→357			securoside A		
37	7.27	[M-H]^−^		433.1149		433.1135			C_21_H_21_O_10_			433→287, 269			neoengeletin		
45 ^a^	8.30	[M-H]^−^		451.1035		451.1029			C_24_H_19_O_9_			451→341			cinchonain Ib		
46	8.32	[M-H]^−^		227.0717		227.0708			C_14_H_11_O_3_			227→209,185, 183, 159, 157, 143			resveratrol		
47 ^a^	8.35	[M-H]^−^		809.2293		809.2293			C_40_H_41_O_18_			809→767, 663, 633			smilaside G		
48 ^a^	8.36	[M-H]^−^		839.2408		839.2398			C_41_H_43_O_19_			839→797, 693, 663, 517			smilaside J		
49 ^a^	8.38	[M-H]^−^		869.2502		869.2504			C_42_H_45_O_20_			869→827, 693, 675			smilaside L		
50	8.40	[M-H]^−^		777.2248		777.2242			C_36_H_41_O_19_			777→735, 717, 601, 559			(3,6-di-O-feruloyl)-β-d-fructofuranosyl-(3,6-di-O-acetyl)-α-d-glucopyranoside		
51	8.42	[M-H]^−^		819.2354		819.2348			C_38_H_43_O_20_			819→777, 643, 601, 513			smilaside C		
52	8.44	[M-H]^−^		923.2604		923.2610			C_45_H_47_O_21_			923→881, 863, 747, 601, 483			smilaside E		
53	8.45	[M-H]^−^		953.2712		953.2715			C_46_H_49_O_22_			953→911, 777, 735, 717, 289			smilaside B		
54 ^a^	8.48	[M-H]^−^		271.0614		271.0606			C_15_H_11_O_5_			271→177, 151			naringenin		
55	8.52	[M-H]^−^		965.2719		965.2715			C_47_H_49_O_22_			965→923, 905, 789, 747, 483			smilaside D		
56	8.55	[M-H]^−^		995.2829		995.2821			C_48_H_51_O_23_			995→953, 819, 777, 513			smilaside A		

^a^ Compared with reference [3]; ^b^ Identified as new compound; ^c^ Compared with reference standards.

### 2.4. Quantitative Analysis

The newly established analytical method was subsequently applied to determine the six compounds of *Smilacis glabrae*. The target compounds were identified based on comparison of retention time and mass information obtained from UHPLC-ESI-MS analysis of the reference standards. Table 3 showed the content determined for each compound. The results indicated that the amount of most components determined was similar in the five different batches.

**Table 2:** Summary of calibration curves, linear range, LOQ, repeatability, intra-day and inter-day precisions and recoveries for six analytes analyzed with the LC-MS system

Analyte	Linear Range (μg/mL)	Calibration Curve (*n* = 7)	r^2^	LOQ (μg/mL)	Repeatability RSD (%)	Intra-day (RSD, %) (*n* = 6)	Inter-day (RSD, %) (*n* = 3)	Recoveries (*n* = 3)				
								Initial (μg)	Spiked (μg)	Detected (μg)	Recoveries (%)	RSD (%)
Neoastilbin	0.82−32.8	*y* = 8593.3 *x* + 281942	0.9993	0.016	2.37	3.19	3.05	3.470	2.628	5.872	96.27	3.51
									3.284	6.890	101.99	3.16
									3.940	7.085	95.63	1.65
Astilbin	3.10−124.1	*y* = 8921.6 *x* + 16423	0.9991	0.062	1.86	1.03	0.76	13.677	9.932	22.963	97.27	2.57
									12.416	25.468	97.60	3.86
									14.900	27.916	97.69	2.66
Neoisoastilbin	0.33−13.3	*y* = 8299.7 *x* + 165713	0.9988	0.067	1.91	2.43	2.49	1.517	1.064	2.342	90.77	4.73
									1.340	2.426	91.94	3.39
									1.606	3.205	102.65	3.83
Isoastilbin	1.78–71.2	*y* = 8479.3 *x* + 161354	0.9981	0.018	2.15	1.07	0.79	7.188	5.702	12.485	96.86	1.80
									7.128	14.153	98.86	2.83
									8.555	15.011	95.35	2.30
Engeletin	0.86–34.4	*y* = 4620.5 *x* − 107846	0.9992	0.017	1.77	2.00	3.91	4.110	2.756	7.038	102.51	2.14
									3.444	7.300	96.59	1.49
									4.132	8.271	100.39	2.37
Isoengeletin	0.28–11.1	*y* = 4472.8 *x* − 12397	0.9991	0.011	1.94	2.83	2.86	1.237	0.896	2.152	100.95	2.34
									1.120	2.368	100.37	3.09
									1.134	2.506	97.18	4.50

**Table 3:** Contents of the six compounds in different batches of *Smilacis glabrae*.

Analyte	Content (μg/g)				
	Batch 1	Batch 2	Batch 3	Batch 4	Batch 5
Neoastilbin	2173.1	2735.9	2356.9	2537.4	2253.7
Astilbin	8548.2	8996.1	9262.1	10,962.2	9988.6
Neoisoastilbin	948.3	1046.4	971.2	1188.7	1097.3
Isoastilbin	4493.2	4189.5	4257.9	2800.9	3461.3
Engeletin	2587.2	2494.3	2682.1	1821.6	2047.6
Isoengeletin	771.6	727.6	834.9	594.3	488.5

## 3. Experimental Section

### 3.1. Chemicals and Materials

HPLC grade acetonitrile and methanol were purchased from Fisher Chemicals (Fairlawn, NJ, USA). Formic acid of HPLC grade was purchased from Sigma Aldrich (St. Louis, MO, USA). Water (18.2 MΩ) was from a Milli-Q water system (Millipore, Bedford, MA, USA). Neoastilbin (**30**), astilbin (**31**), neoisoastilbin **(34**), isoastilbin (**35**), engeletin (**38**) and isoengeletin (**42**) were provided by Dr. Lixiong from the Guangdong Provincial Hospital of Chinese Medicine. Three batches of *Smilacis glabrae* originating from Guangdong Province, China were supplied by Kangmei Pharmaceutical Co. Ltd. (Puning, China). Two batches of *Smilacis glabrae* from the Hunan and Guangxi provinces of China were purchased from Er-tian-tang Pharmacy (Guangzhou, China). Voucher samples were deposited in the Laboratory of Chinese Materia Medica Preparation, Second Affiliated Hospital, Guangzhou University of Traditional Chinese Medicine.

### 3.2. Standard Solutions and Sample Preparation

The standard solution mixture of the six flavonoids was prepared by dissolving the reference substances in methanol to final concentration of 32.8 μg/mL for neoastilbin, 124.1 μg/mL for astilbin, 13.3 μg/mL for neoisoastilbin, 71.2 μg/mL for isoastilbin, 34.4 μg/mL for engeletin and 11.1 μg/mL for isoengeletin, respectively. Then, the standard solution mixture was diluted to 80%, 60%, 40%, 20%, 10%, 5% and 2.5% of the concentration of the original solution. All the standard solutions were stored at 4 °C.

The dried rhizome (0.2 g, 60 mesh) was accurately weighed and ultrasonically extracted by infusion with 25 mL water for 30 min. The extracted solution was centrifuged at 10,000 rpm for 10 min, and then filtered through a 0.22 m nylon membrane filter prior to injection for UHPLC-MS analysis.

### 3.3. Analytical System

Chromatographic separation was performed on an Accela™ ultra high pressure liquid chromatography (UHPLC) system (Thermo Fisher Scientific, San Jose, CA, USA) comprising a UHPLC pump, a PDA detector, scanning from 200 to 400 nm, and an autosampler settled to 30 °C. The LC conditions were as follows: column: Agilent Eclipse Plus C18 (100 mm × 3.0 mm, 1.7 μm); mobile phase: acetonitrile (A) and water (B) both containing 0.1% (v/v) formic acid; gradient: 0 min, 10: 90; 1 min, 20: 80; 3–6.5 min, 23: 77; 7 min, 80: 20; 9–10 min, 100: 0 (A: B, v/v); flow rate: 0.3 mL/min; injection volume: 10 μL.

### 3.4. Qualitative Characteristic of Chemical Constituents

Identification of chemical constituents in *Smilacis glabrae* extract was performed by UHPLC-ESI-MS^n^ analysis. MS analysis was performed using an LTQ Orbitrap^XL^ hybrid mass spectrometer (Thermo Fisher Scientific), fitted with an ESI source, and operated in negative ion mode, with a mass range of 100–1500 with resolution set at 30000 using the normal scan rate.

The data-dependent MS/MS events were always performed on the most intense ions detected in full scan MS. The MS/MS isolation width was 1 amu, and the normalized collision energy was 35% for all compounds. Nitrogen was used as sheath gas and helium served as the collision gas. The key optimized ESI parameters were as follows: source voltage: 3.8 kV; sheath gas (nitrogen): 50 L/min; auxiliary gas flow: 10 L/min; capillary voltage: –35.0 V; capillary temperature: 300.0 °C; tube lens: –110.0 V. The ion injection time used was 50.0 ms. MS scan functions and HPLC solvent gradients were controlled by the Xcalibur data system (Thermo Fisher Scientific). Data was collected and analyzed with Xcalibur 2.0.7 software (Thermo Fisher Scientific). The Orbitrap mass analyzer was calibrated according to the manufacturer’s directions using a mixture of caffeine, methionine-arginine-phenylalanine-alanine-acetate (MRFA), sodium dodecyl sulfate, sodium taurocholate and Ultramark 1621 in an acetonitrile-methanol-water solution containing 1% acetic acid by direct injection at a flow rate of 5 μL/min in negative mode before analysis.

### 3.5. Validation of the Quantitative Analysis

A calibration curve was used to determine the calculated concentration of the samples. The calibration curve of each compound was performed with at least six appropriate concentrations. The limit of quantification (LOQ) under the present chromatographic conditions was determined at signal-to-noise ratios (S/N) of 10.

Intra- and inter-day variations were chosen to determine the precision of the developed method. The precision was examined by five repetitive injections in the same day and in three consecutive days, respectively. The relative standard deviation (R.S.D.) was considered as the measure of precision.

The accuracy was evaluated by calculating the mean recoveries of six reference standards from the spiked standard solutions. A known amount of *Smilacis glabrae* sample was spiked with the standard solution at three different concentration levels. The high spiked amount was 1.2 times of the known amount sample, the middle spiked amount was 1.0 times of the known amount sample and the low spiked amount was 0.8 times of the known amount sample. The recovery percentages were calculated using to the following equation: (total detected amount – original amount)/added amount ×100%.

## 4. Conclusions

In this study, a total of 56 compounds, including six minor new ones, were simultaneously detected and identified by UHPLC-LTQ-Orbitrap-MS. Based on the qualitative analysis, a rapid method was established for quantitative analysis of six marker components in *Smilacis glabrae* extract. This is the first report on the comprehensive determination of chemical constituents in *S. glabrae* by UHPLC-LTQ-Orbitrap-MS. The results would provide the chemical support for the further pharmacokinetic studies and for the improvement of quality control of *Smilacis glabrae* and its preparations. The study also suggested that UHPLC-ESI/LTQ-Orbitrap mass spectrometry would be a powerful and reliable analytical tool for the characterization of chemical profile in complex chemical system, such as TCM preparations.

## Supplementary Materials

Supplementary materials can be accessed at: http://www.mdpi.com/1420-3049/19/7/10427/s1.
