# Supplementary material for: Qualitative and Quantitative Analysis of Rhizoma Smilacis glabrae by Ultra High Performance Liquid Chromatography Coupled with LTQ OrbitrapXL Hybrid Mass Spectrometry
Source: Molecules. 2014 Jul 17;19(7):10427–39. doi: 10.3390/molecules190710427 (PMC6270682; doi:10.3390/molecules190710427)

Supplementary Information

Figure S1. The chemical structures of identified compounds of *Smilacis glabrae* extract.

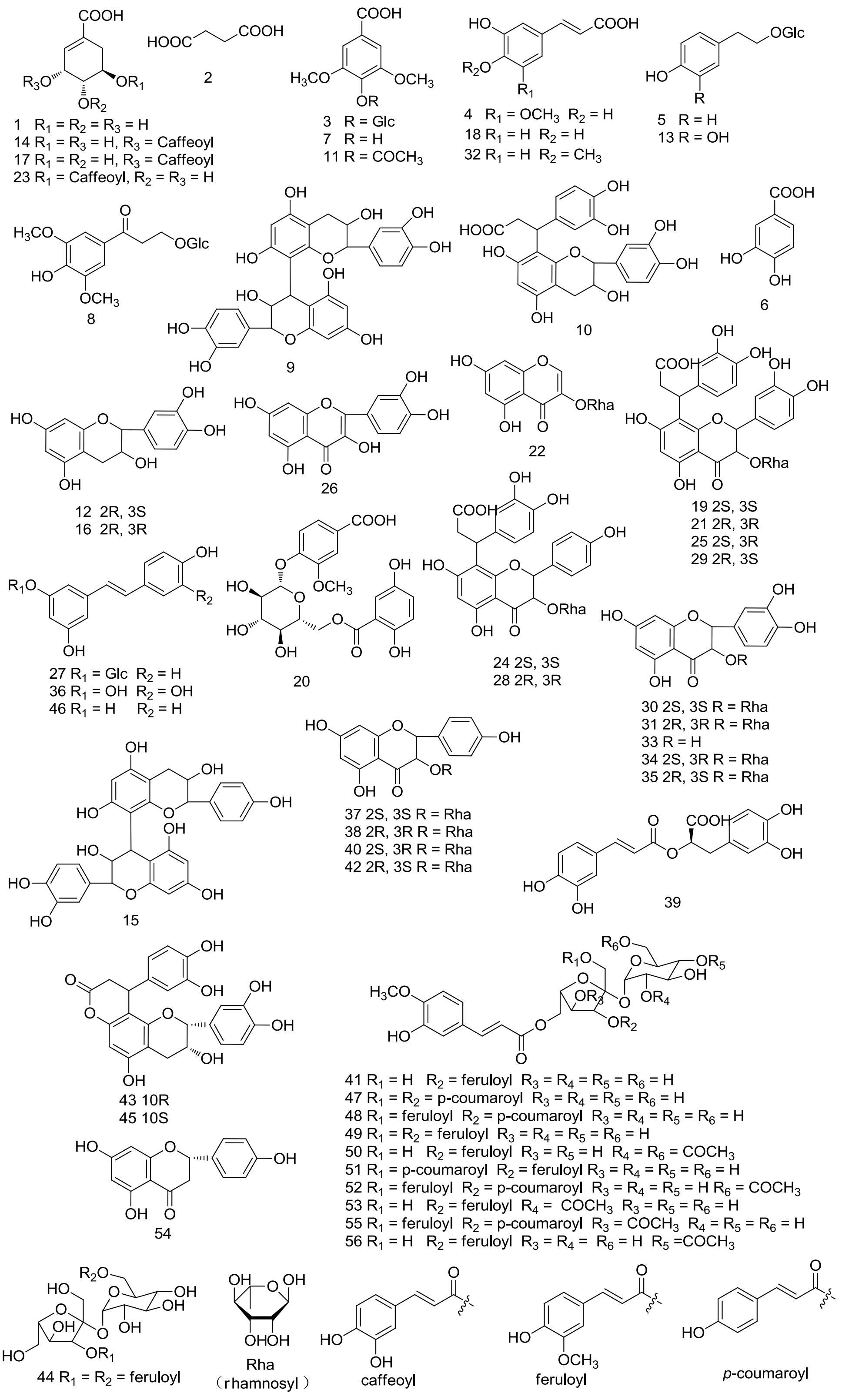

Figure S2. UV spectra of compounds **30**, **31**, **34**, **35 and 19**, **21**, **25**, **29**.

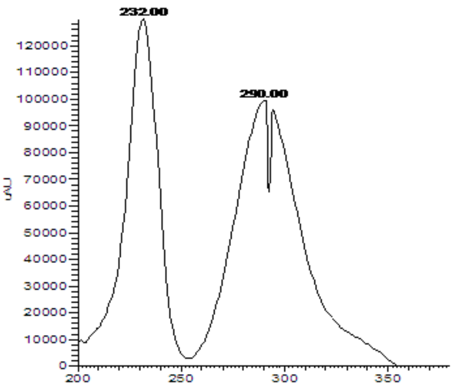

UV spectrum of compound **30**

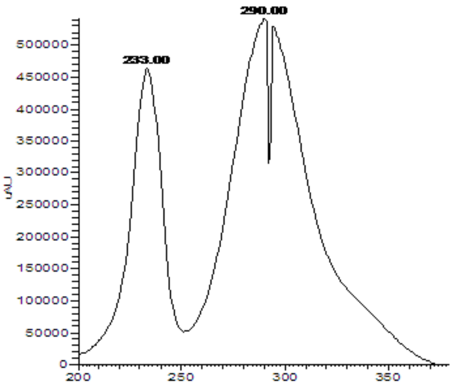

UV spectrum of compound **31**

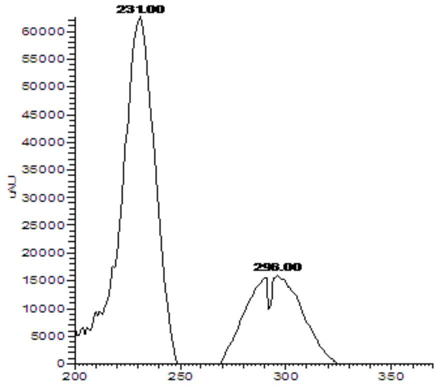

UV spectrum of compound **34**

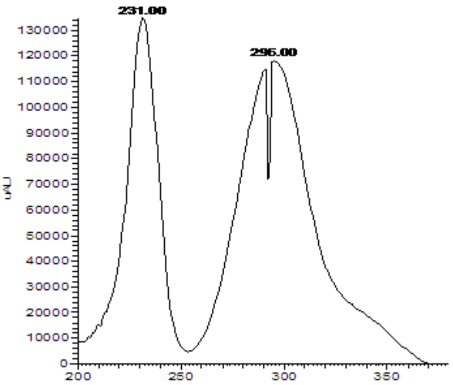

UV spectrum of compound **35**

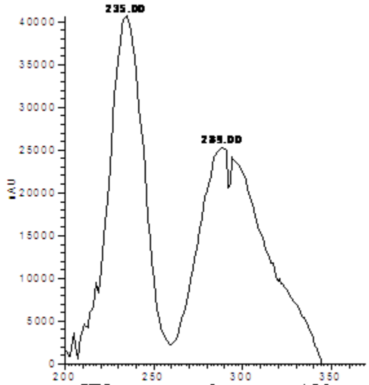

UV spectrum of compound **19**

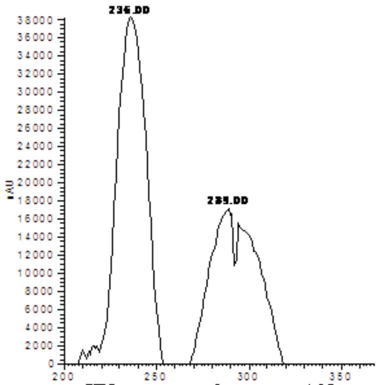

UV spectrum of compound **21**

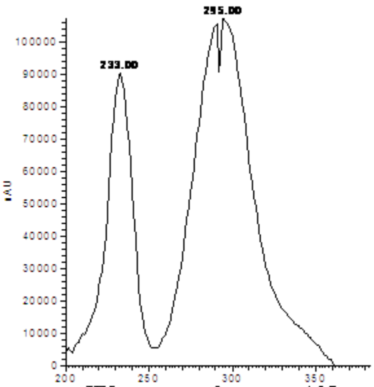

UV spectrum of compound **25**

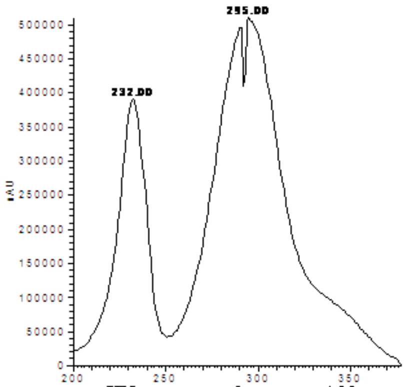

UV spectrum of compound **29**

Figure S3. Proposed fragmentation pathway for compounds **24** and **28**.

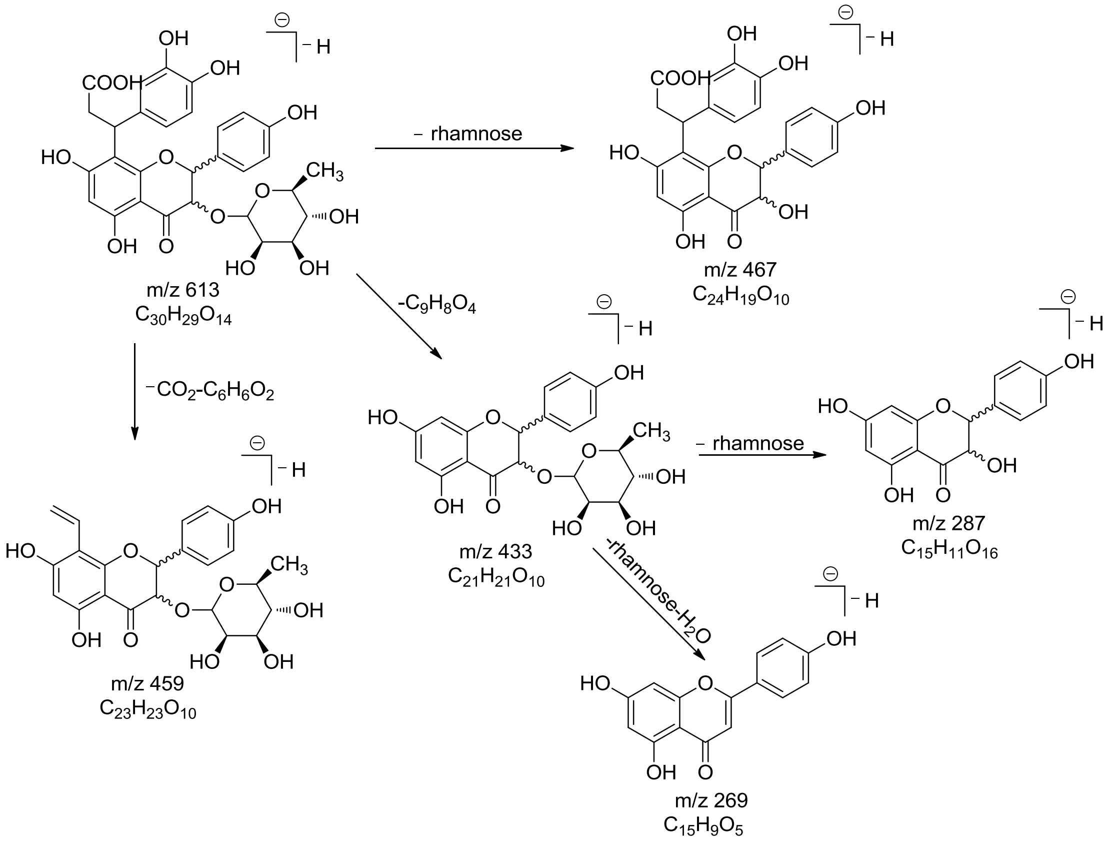

Supplement: Supplementary file 1 [file molecules-19-10427-s001.pdf]
